# Lipoprotein (a) in type 2 diabetes mellitus: Relation to LDL:HDL ratio and glycemic control

**DOI:** 10.4103/0973-3930.53125

**Published:** 2009

**Authors:** Seema Singla, Kiranjeet Kaur, Gurdeep Kaur, Habir Kaur, Jasbinder Kaur, Shivani Jaswal

**Affiliations:** Department of Biochemistry, Govt Medical College, Chandigarh, India; 1 Department of Biochemistry, Govt Medical College, Patiala, India; 2 Department of Medicine, Govt Medical College, Patiala, India

**Keywords:** Type 2 diabetes, lipoprotein(a), LDL: HDL ratio, glycated hemoglobin

## Abstract

**BACKGROUND:**

Increased lipoprotein (a) [Lp (a)] concentrations are predictive of coronary artery disease (CAD). Type 2 diabetes mellitus also leads to dyslipidemia, like elevated triglyceride levels and low HDL levels, which are known risk factors for CAD. This study was designed to investigate the levels of Lp (a) in type 2 diabetic patients and their association with LDL: HDL ratio and glycemic control.

**MATERIALS AND METHODS:**

The study included 60 patients of type 2 diabetes and 50 age and sex matched controls. The Lp(a) levels in the diabetic group were compared with the control group and the relationship between the Lp(a) levels and LDL: HDL ratio was evaluated. Diabetic group was further divided into three subgroups according to levels of glycated hemoglobin. Lp(a) levels and glycated hemoglobin in controlled and uncontrolled diabetes mellitus were also compared to find out any correlation between them. Statistical analysis was done using the students ‘t’ test and Chi square test.

**RESULTS:**

Lp(a) levels were found to be significantly increased in the diabetic group as compared to the control group (*P*< 0.001). LDL: HDL ratio was also increased in the diabetic group as compared to the control group. Lp(a) levels showed no association with LDL: HDL ratio and degree of glycemic control in these patients.

**CONCLUSIONS:**

The results of the present study suggest that Lp(a) levels are increased in type 2 diabetic patients. The elevated Lp(a) levels do not reflect the glycemic status and are also independent of increase in LDL:HDL ratio suggesting different metabolic pathways and the genetic connection for LDL and Lp(a).

## Introduction

The risk of cardiovascular disease in type 2 diabetic subjects is increased two to four folds over age matched non-diabetic subjects.[1] As compared to the non-diabetic controls, type 2 diabetic subjects have increased triglycerides levels and decreased high-density lipoprotein cholesterol (HDL), but relatively small differences in low-density lipoprotein cholesterol (LDL). The excess risk in the diabetic subjects is only partially explained by the standard risk factors measured in these subjects.[2]

Considerable data has suggested that Lp(a) is a major risk factor for cardiovascular disease.[3] Lipoprotein (a) was first identified as a distinct lipoprotein particle in 1963 by Berg.[4] Lp(a) is an unusual serum lipoprotein characterized by the presence of a unique glycoprotein (a) linked to apoprotein B-100 by disulphide linkages. The lipoprotein (a) obtained after reduction is virtually identical to LDL in its physicochemical properties and its cellular uptake by the LDL receptors in cultured human fibroblasts. However, native Lp(a) is a poor ligand for LDL receptors and dietary changes and drugs that alter LDL levels do not affect Lp(a).[5] The amino acid sequence of apoprotein(a) is found remarkably similar to that of human plasminogen. This striking homology has given rise to the hypothesis that the increased risk of premature atherosclerosis and thrombotic diseases associated with elevated Lp(a) levels rises from molecular mimicry of plasminogen by apo(a).[6]

Studies regarding association of the mean Lp(a) levels with diabetes is contradictory. Arauz *et al*.[7] found a higher mean concentration of Lp(a) in a combined group of type 1 and type 2 diabetic subjects but found no association of glycated hemoglobin with Lp(a) in type 2 subjects. In one study of well controlled type 2 diabetics, the Lp(a) concentration was actually lower in diabetics than in the non-diabetic control subjects.[8] Chico *et al*.[9] found no difference in the mean Lp(a) concentration between diabetic and non-diabetic subjects. The possible association of the Lp(a) levels and the metabolic control is of major interest since the serum concentration of apoprotien(a), the unique protein of Lp(a), may be genetically controlled to a large degree.[2]

The present study aimed to evaluate the association of the Lp(a) levels with type 2 diabetes mellitus in addition to its association with the degree of glycemic control and LDL: HDL ratio.

## Materials and Methods

The study was conducted in the Rajindra Hospital, Patiala, India. The subjects included 60 patients with insidious onset of type 2 diabetes mellitus attending the medical clinics. Patients with history of angina, ketoacidosis, myocardial infarction, abnormal thyroid and liver function tests were excluded from the study.

Diabetes mellitus was diagnosed according to American Diabetes Association (ADA) criteria 2000.[10] (Fasting glucose level ≥126mg/dl and 2-hour post prandial ≥200mg/dl). The subjects who did not meet ADA criteria, but were under treatment with oral hypoglycemic agents or insulin were also considered to be diabetic. Duration of diabetes and anti-diabetic therapy was also noted. The control group comprised of 50 healthy age and sex matched subjects.

The Lp(a) levels were estimated by ELISA.(CV < 7%) using a commercially available kit supplied by HYPHEN Biomed, France. HDL, serum cholesterol and serum triglycerides were estimated using commercially available enzymatic kits, while the LDL was calculated using Frieldwald's formula. The glycated hemoglobin (HbA1c) was estimated by ion exchange chromatography method.

For statistical analysis, the control group was compared with the diabetic group. The diabetic group was further divided into subgroups A, B and C depending upon their HbA1c levels. Group A comprised of patients who had HbA1c ≤ 6%, Group B with HbA1c between 6.1-8% and Group C with HbA1c ≥ 8.1%. The statistical analysis was done between A and B, B and C, A and C.

## Results

There were 32 mals (Age 52.3 ± 8.17 years) in the diabetic group (N = 60) and 26 mals (Age 54.12 ± 8.17 years) in the control group (N = 50).

The Lp(a) and the LDL levels were significantly increased in the diabetic group as compared to the control group (*P*< 0.001 and *P*<0.05 respectively).

**Figure 1:**
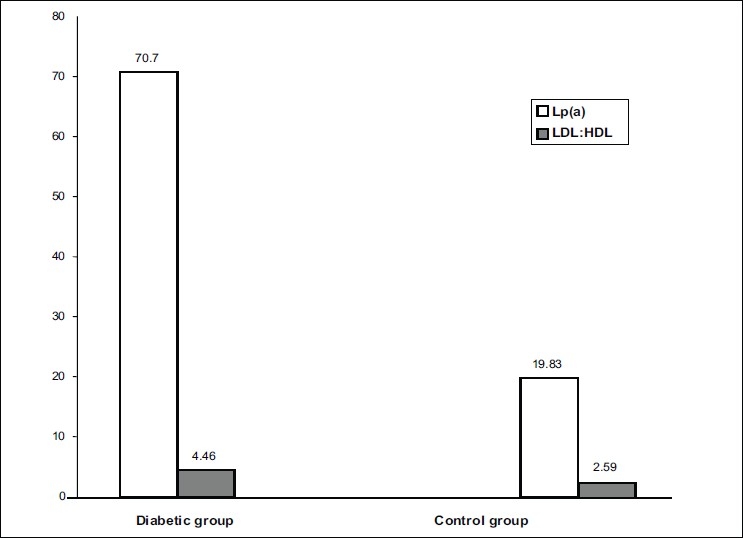
Lp(a) & LDL:HDL ratio in Diabetic and Control Groups

Mean HDL levels were low in the diabetic group as compared to the control group and this difference was statistically significant (*P*<0.001). The LDL: HDL ratio was also significantly higher in the diabetic group [Figure 1].

The mean Lp(a) levels and LDL: HDL ratio in Group A (HbA1c ≤ 6%), B (HbA1c 6.1-8%) and C (HbA1c ≥ 8.1%) are shown in Table 1. When Lp (a) levels were compared between these three subgroups, the result was non-significant (*P*> 0.05). Statistical analysis of LDL: HDL ratio between subgroups A and B, B and C and C and A was also found to be non-significant (*P*> 0.05).

**Table 1:** HbA1c, LDL: HDL ratio and Lp(a) in Subgroups of Diabetic group

Subgroups	Mean HbA1c Level %		LDL:HDL ratio		Mean Lp(a) mg/dl
Group A n=10	≤ 6%		4.48 ± 0.99		60.94 ± 27.25
Group B n=27	6.1-8%		4.48 ± 0.95		73.47 ± 43.96
Group C n=23	≥ 8.1%		4.43 ± 1.14		71.16 ± 48.43

Mean Age and Sexwise distribution of subjects in the Diabetic and Control groups					

	Diabetic group (n = 60)		Control group (n = 50)		Significance
			
Sex	Males	Females	Males	Females	*P* > 0.05
	32	28	26	24	NS
Age	52.73 ± 8.17 years		54.12 ± 8.18 years		*P* > 0.05 NS

**Figure 2:**
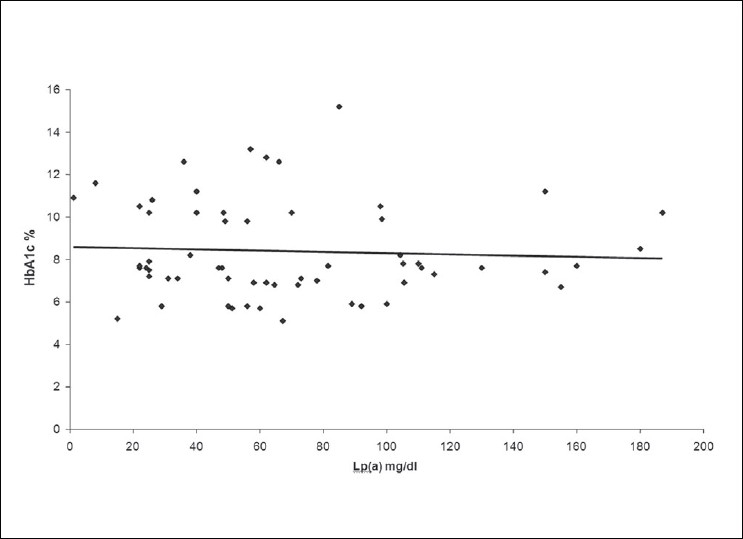
Correlation between Lp(a) and HbA1c in Diabetic Group

**Figure 3:**
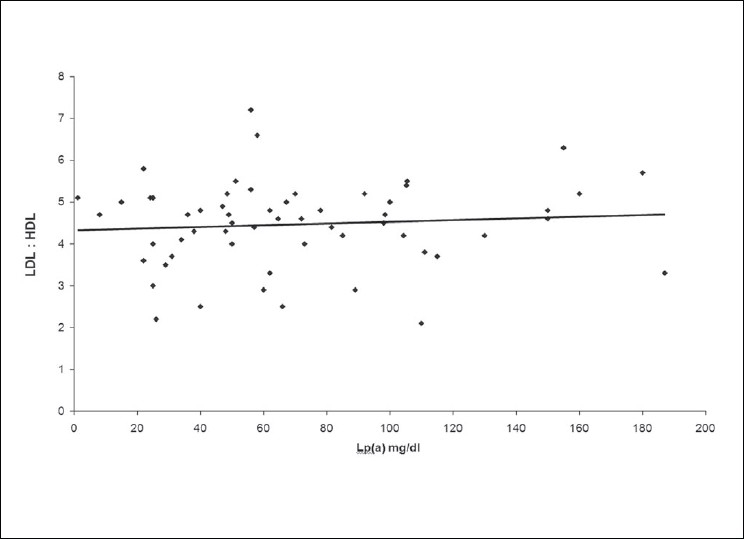
Correlation between Lp(a) and LDL: HDL ratio in Diabetic group

The correlations between the levels of Lp (a) and HbA1c and between the Lp (a) levels and LDL: HDL ratio have been depicted in Figures 2 and 3 respectively. No significant correlation was found between the Lp (a) and the HbA1c levels (*P*>0.05, r = -0.01) and Lp (a) and LDL: HDL ratio (*P* >0.05, r = 0.08)).

## Discussion

Diabetes mellitus comprises of a group of disorders that share a phenotype of hyperglycemia. The complications are an important cause of morbidity and mortality in the diabetic patients. These complications are a result of interaction of multiple metabolic, genetic and other factors.[11]

Atherosclerosis is one of the common complications of diabetes and abnormal lipoprotein metabolism may account for the increased frequency of atherosclerotic lesion in diabetics. Large data shows an increased incidence of CAD with increased Lp(a) levels. Gambhir *et al*.[12] found that low HDL (*P*<0.015) and high Lp(a) (*P*<0.001) were independent risk factors for premature coronary artery disease below 40 years of age. Solfrizzi *et al*.,[13] however, suggested that elevated Lp(a) levels did not appear to be an independent predictor of CAD but is a risk factor only in subjects with type 2 diabetes mellitus and elevated LDL levels.

Lp(a) interferes with the fibrinolytic function of plasminogen, thereby promoting the thrombotic events. It inhibits tissue plasminogen activator (t-PA) binding to fibrin and also suppresses the fibrin and fibrinogen fragment dependent enhancement of plasminogen activation by t-PA in some assay systems.[14] Lp(a) also inhibits plasminogen activation by streptokinase.[15] It has been shown to compete for the binding of plasminogen to monocytoid cell and epithelial cells.[16] Tetranectin, a plasma protein, binds reversibily to plasminogen and enhances plasminogen activation by t-PA. Lp(a) was found to bind to tetranectin with higher affinity than Glu or Lys plasminogen.[17] Systemic atherosclerosis measured as the peripheral occlusive arterial disease is strongly associated with serum Lp(a) in both type 1 and type 2 diabetes mellitus.[18] The Lp(a) levels have also been suggested to play a pathogenic role in development of complications like gangrenous foot lesions in patients of diabetes mellitus.[19]

The present study was conducted to evaluate the Lp(a) levels in the patients of type 2 diabetes mellitus and to find out its association, if any, with the LDL:HDL ratio and HbA1c in these patients. The results suggest a strong association between the Lp(a) levels and the type 2 diabetes mellitus, as the levels of Lp(a) were found to be significantly higher in the study group as compared to the controls. Our results correlate with the findings forwarded by Habib and Aslam.[20]

Type 2 diabetes mellitus has a strong genetic component.[21] The genetic basis of dyslipidemia has also been well established.[22] The genetic predisposition of both the type 2 diabetes mellitus and the deranged Lp(a) levels may be a common basis of the two events occurring together. Lp (a) concentrations in serum is also affected by apo (a) phenotypes. Uterman *et al*.[23] categorized Lp(a) patterns into phenotypes F,B,S1,S2, S3 and S4 according to their electrophoretic mobilities as compared to apo B-100. Family studies are compatible with the fact that Lp(a) glycoprotein phenotypes are controlled by a number of autosomal alleles at a single locus. Comparison of Lp(a) levels in different phenotypes revealed a highly significant association of phenotype with concentration. Phenotypes B,S1 and S2 are associated with high and phenotypes S3 and S4 with low plasma concentration. It may be possible that increased Lp(a) concentrations in diabetics is due to the presence of apo(a) phenotypes associated with increased Lp(a) concentrations in these patients. Shi *et al*.[24] found that in comparison with the diabetics without complications, apo(a) phenotypes differed significantly in patients with nephropathy, hypertension, coronary heart disease and myocardial infarction. However, Hirata *et al*.[25] suggested that Lp(a) levels in diabetic patients are not regulated by smaller apo(a) isoforms and that serum Lp(a) levels are greater in diabetic patients than in non-diabetic family members even when they share same phenotype.

Heller *et al*.[26] suggested that hyperinsulinemia can be the causal factor for increase in the Lp(a) levels in type 2 diabetics. Similar results have also been reported by Wolffenbuttel *et al*.[27] They reported that Lp(a) levels were elevated in diabetics as compared to non-diabetic subjects of similar age but did not change with insulin and there was no correlation with the degree of metabolic control and changes in Lp(a) levels. However, Alagozlu *et al*.[28] reported that Lp(a) levels in particular are decreased by insulin or sulfonylurea in non-obese patients with type 2 diabetes mellitus.

Metabolic reasons for lower HDL levels have not been fully documented. Decreased synthesis of HDL has been found in one small study.[29] Increased clearance of HDL particles from the plasma space may also be operative particularly in patients with hypertriglyceridemia.[30] Schmitt *et al*.[31] suggested that LDL uptake by fibroblasts may be impaired in type 2 diabetics. This leads to increase in LDL: HDL ratio in type 2 diabetics. In our study, LDL: HDL ratio did not differ significantly between controlled and uncontrolled diabetics (*P*>0.05). Similar results have also been forwarded by Haffner *et al*.[2] However, Schmitt *et al*.[31] found that LDL: HDL ratio correlated with HbA1c better than any of the lipids or lipoprotein fractions. LDL: HDL ratio changed significantly than did its component fractions. We also did not find any significant association between the Lp(a) levels and LDL:HDL ratio. Ramirez *et al*.[8] also found no significant correlation between Lp(a) and LDL levels, suggesting that the Lp(a) and the LDL levels are under different metabolic control.

In our study we found that the mean Lp(a) levels did not vary significantly between subgroups A, B and C of the diabetic group divided on the basis of HbA1c levels. This suggests that degree of glycemic control does not affect Lp(a) levels. Similar results were also found in another study from our subcontinent.[20] Thus, the effect of glycemic control on Lp(a) levels is small in type 2 diabetics. As higher Lp(a) levels are found to be associated with diabetic complications[3,19] lowering of Lp(a) levels by life style modifications and drugs should be considered. However, alteration of the Lp(a) levels with lipid lowering drugs is difficult. Neomycin and nicotinic acid have been reported to lower Lp(a) concentrations[32] while cholestyramine has little effect.[33] Weight loss has also been found to lower the Lp(a).[34] After 4 weeks of weight loss, the Lp(a) is decreased by 19% in men and 30% in premenopausal women, although no relationship of adiposity to Lp(a) concentration was observed.

From the present study we can conclude that type 2 diabetes mellitus is strongly associated with increased Lp(a) levels. We did not find any significant correlation between the levels of Lp(a) and glycated hemoglobin. No significant correlation could be established between Lp(a) and LDL: HDL ratio. However, as the present study was performed on a population confined to a particular area, the results do not necessarily apply to the other racial groups. The small sample size is also another limitation of our study. Further prospective studies will be required to establish these findings.
